# Supplementary material for: Impact of Glass Composition on Hydrolytic Degradation of Polylactide/Bioactive Glass Composites
Source: Materials (Basel). 2021 Feb 1;14(3):667. doi: 10.3390/ma14030667 (PMC7867177; doi:10.3390/ma14030667)
Supplement: Supplementary file 1 [file materials-14-00667-s001.zip › materials-1063455-supplementary final.pdf]

materials
MDPI

Supplementary Materials

Impact of Glass Composition on Hydrolytic Degradation of Polylactide/Bioactive Glass Composites

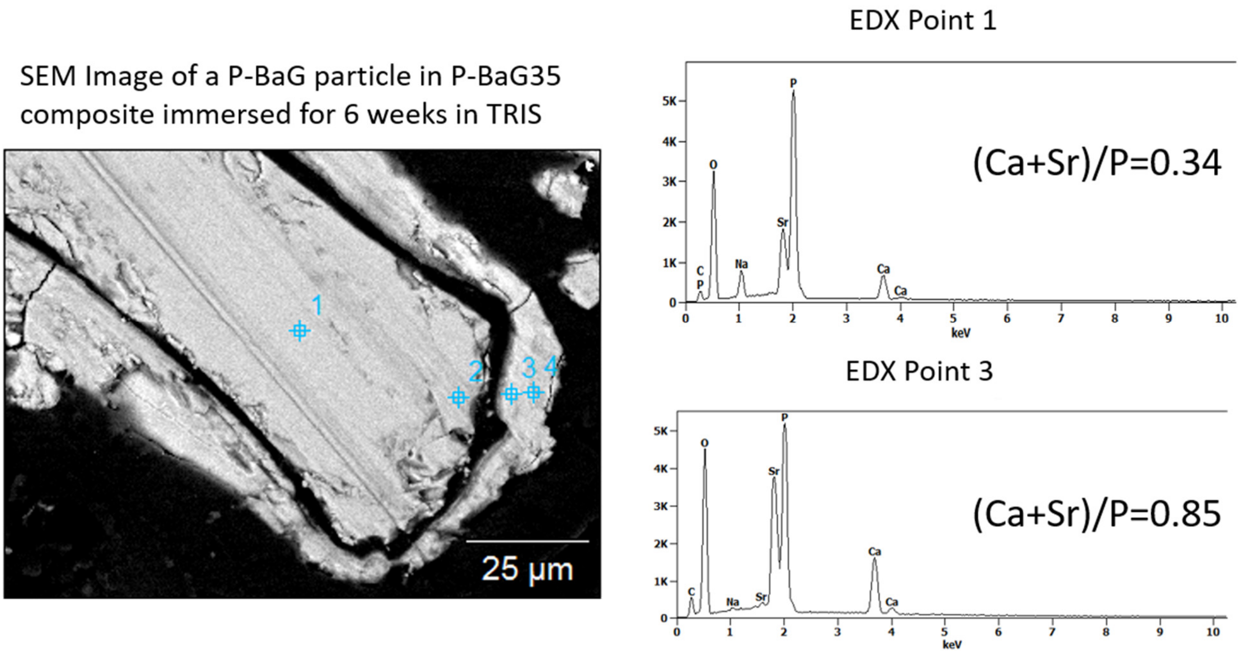

Figure S1. SEM image of a phosphate glass particle in the P-BaG35 composites immersed for six weeks in TRIS buffer solution along with the EDX spectrum of point 1 (unreacted glass) and point 3 (reactive layer).

The SEM image, in Figure S1, exhibits a partially reacted phosphate glass particle. The core of the particle corresponds to the unreacted glass, showing a (Ca+Sr)/P ratio of 0.34, in agreement with the theoretical glass composition. Around the unreacted glass a reactive layer has precipitated with a (Ca+Sr)/P ratio of 0.85 similar to the values reported in Table 4 and corresponding to the precipitation of dicalcium phosphate di-hydrate.
